# New Insights in Anorexia Nervosa

**DOI:** 10.3389/fnins.2016.00256

**Published:** 2016-06-29

**Authors:** Philip Gorwood, Corinne Blanchet-Collet, Nicolas Chartrel, Jeanne Duclos, Pierre Dechelotte, Mouna Hanachi, Serguei Fetissov, Nathalie Godart, Jean-Claude Melchior, Nicolas Ramoz, Carole Rovere-Jovene, Virginie Tolle, Odile Viltart, Jacques Epelbaum

**Affiliations:** ^1^Centre Hospitalier Sainte-Anne (CMME)Paris, France; ^2^UMR-S 894, Institut National de la Santé et de la Recherche Médicale, Centre de Psychiatrie et NeurosciencesParis, France; ^3^Université Paris Descartes, Sorbonne Paris CitéParis, France; ^4^CB Maison de Solenn-Maison des Adolescents, Cochin HospitalParis, France; ^5^Institut National de la Santé et de la Recherche Médicale U982, Laboratory of Neuronal and Neuroendocrine Differentiation and Communication, Institute for Research and Innovation in BiomedicineRouen, France; ^6^Normandy UniversityCaen, France; ^7^University of RouenRouen, France; ^8^Adolescents and Young Adults Psychiatry Department, Institut Mutualiste MontsourisParis, France; ^9^CESP, Institut National de la Santé et de la Recherche Médicale, Université Paris-Descartes, USPCParis, France; ^10^University Reims, Champagne-Ardenne, Laboratoire Cognition, Santé, Socialisation (C2S)-EA 6291Reims, France; ^11^Institut National de la Santé et de la Recherche Médicale U1073 IRIB Normandy UniversityRouen, France; ^12^Faculté de Médecine-PharmacieRouen, France; ^13^Université de Versailles Saint-Quentin-en-Yvelines, Institut National de la Santé et de la Recherche Médicale U1179, équipe Thérapeutiques Innovantes et Technologies Appliquées aux Troubles Neuromoteurs, UFR des Sciences de la Santé Simone VeilMontigny-le-Bretonneux, France; ^14^Département de Médecine (Unité de Nutrition), Hôpital Raymond Poincaré, Assistance Publique-Hôpitaux de ParisGarches, France; ^15^Institut de Pharmacologie Moléculaire et Cellulaire, UMR6097, Centre National de la Recherche ScientifiqueValbonne, France; ^16^Université Lille, Inserm, CHU Lille, UMR-S 1172 - JPArc - Centre de Recherche Jean-Pierre AUBERT Neurosciences et CancerLille, France

**Keywords:** eating disorders, reward system adaptations, microbiota, autoantibodies, susceptibility factors, mental homeostasis

## Abstract

Anorexia nervosa (AN) is classically defined as a condition in which an abnormally low body weight is associated with an intense fear of gaining weight and distorted cognitions regarding weight, shape, and drive for thinness. This article reviews recent evidences from physiology, genetics, epigenetics, and brain imaging which allow to consider AN as an abnormality of reward pathways or an attempt to preserve mental homeostasis. Special emphasis is put on ghrelino-resistance and the importance of orexigenic peptides of the lateral hypothalamus, the gut microbiota and a dysimmune disorder of neuropeptide signaling. Physiological processes, secondary to underlying, and premorbid vulnerability factors—the “pondero-nutritional-feeding basements”- are also discussed.

## Introduction

Anorexia nervosa (AN) is an eating disorder defined as an abnormally low body weight associated with intense fear of gaining weight and distorted cognitions regarding weight, shape, and drive for thinness (American Psychiatric Association, 2013). This disorder has a 12-month prevalence rate of 0.4% among females, and is characterized by the highest mortality rate of all psychiatric disorders (Harris and Barraclough, 1998) and exceptionally high relapse rates (Zipfel et al., 2000).

The research on anorexia nervosa has historically focused on pituitary glands (Gull, 1888), psychiatric aspects associated with a minimum of 12 kg voluntary loss (Bliss and Branch, 1960), or hormones such as oestradiol, progesterone, and LHRH (Boyar et al., 1974), but the aetiology of AN is still unknown, while its development, progression and outcome are considered as clearly influenced by biological, sociocultural, and psychological factors. At the biological level, increased levels of AgRP, NPY, and Ghrelin were, for example, considered as driving the rewarding aspects of thinness, while decreased levels of BDNF, Oxytocin, TRH, VP, Leptin, and PYY have been related to the abnormal satiety feedback observed in AN (Tortorella et al., 2014). But with the progress of brain imaging techniques (especially functional MRI), GWAS and epigenetic approaches, animal models, discovery of neuropeptides (such as 26RFa), and increased knowledge in the role of gut microbiota, it now appears possible to propose more comprehensive models of AN, which also take into account the improved knowledge of psychological risk factors during (or even before) childhood, and the new insights given by the high psychiatric co-morbidity rate of AN. The present review collection proposes seven short contributions, based on these different approaches, in order to propose a tentatively holistic model of AN, based on the concept of homeostasis disruption both at the level of the body (neuroimmunoendocrine approaches), the brain (imaging), and the mind (psychological and clinical approaches).

## Model 1: anorexia nervosa is an abnormality of reward pathway: evidences from physiology, genetics, epigenetics, and brain imaging

Since three decades, physiological evidences support the hypothesis that anorexia nervosa (AN) can be considered as a starvation addiction, driven by abnormalites of the food reward pathway. Novel tools from molecular genetics and brain imaging supplied more evidence supporting this pathophysiological hypothesis.

Numerous opioid neuropeptides have been identified and characterized as being involved in the regulation of vital functions, such as appetite and reproduction, conferring them reward properties, apparently as highly addictive as the exogenous opiates (Le Merrer et al., 2009). The addiction theory in eating disorders is therefore supported by the fact that both appetite dysfunction (starvation and bingeing) and intense physical activity stimulate endorphin activity in 80% of AN patients (Kaye et al., 1989). In 1982, Kaye et al. reported that the opioid activity from the cerebrospinal fluid was significantly higher in underweight AN patients compared to controls. In contrast, this activity decreases in AN patients with a restored weight or in recovered cases compared to controls (Kaye et al., 1982). Furthermore, the plasma levels of codeine and morphine were reported as significantly elevated in AN patients compared to a control group (Marrazzi et al., 1997). These endogenous opioids could be released during the first diets, the feedback control of opioids then reinforcing the associated starvation process in some at-risk subjects. Indeed, peripheral endorphins may foster survival in starvation conditions by conservation of nutrients and water and by decreasing energy-expending activities (Margules, 1979).

Functional brain imaging study analyzing a simple monetary reward task has shown that healthy women had different striatal activity for positive vs. negative feedbacks, while recovered AN patients did not (Wagner et al., 2007). This study supported the hypothesis of an altered reward process in AN. Another functional brain imaging study demonstrated the existence of an increased salience attribution to rewarding and aversive food stimuli in recovered AN patients (Cowdrey et al., 2011). Structural brain imaging also disclose alteration in the brain regions that are involved in reward circuitry in AN patients, and even in recovered ones (Frank et al., 2013). Recent evidence more precisely showed that patients with AN differ from controls as they favor delayed rewards (larger and later instead of smaller and sooner monetary rewards), being the only one to have an increased activity of the ventral striatum for such rewards (Decker et al., 2015). Finally, two functional brain imaging studies based on the evaluation of visual stimuli depicting a female body with underweight, normal weight, and overweight canonical whole-body features, assessed brain activity with a “feel” task (in a self-referring way) and a “weight” task, as a control task (Fladung et al., 2010, 2013). No difference between AN patients and controls was reported for the “weight” task. In contrast, opposite score for the “feel” task was observed for both adult and adolescent AN patients compared to healthy subjects. Most interestingly, the functional activity of the ventral striatum was significantly higher in AN patients for underweight features (compared to controls) and dramatically reduced for normal body features in adults (Fladung et al., 2010). Similar trends were observed when adolescent were analyzed (Fladung et al., 2013). These studies support once again the existence of an alteration of the reward circuitry in AN, likely due to a reinforcing effect of starvation, due to an increased hedonic feeling of underweight, and a decreased positive feeling of normal bodyweight. Another candidate to explain abnormal reward process is excessive exercise, which is observed in 80% of patients with AN (Davis et al., 1997). In the largest GWAS performed up to now on AN, two SNPs were marginally associated with the disorder (*p* = 5 × 10^−6^), one of them (rs17030795) being located in PPP3CA, a calcineurin gene which might be involved in human variations in endurance exercise capacity and tolerance (He et al., 2010b). Furthermore, in a rodent study, leptin was influencing the motivational effects of running via dopamine tone (Fernandes et al., 2015), leptin being more depending of DNA methylation (i.e., under epigenetic regulation) rather than direct genetic control (Tremolizzo et al., 2014).

Interestingly, one of the first AN genome-wide linkage study, performed in 37 AN families, identified a significant peak on chromosome 1p33-36, containing *HTR1D* and *OPRD1* genes, respectively encoding for 1D serotonin receptor and opioid delta receptor. Furthermore, individual variants and haplotypes within both *HTR1D* and *OPRD1* genes were associated with AN in a candidate gene study performed on 191 AN patients and 98 controls (Bergen et al., 2003). The role of the *OPRD1* gene in anorexia nervosa was replicated in an independent study comparing 226 AN patients to 678 controls (Brown et al., 2007). Lastly, a genome-wide association study performed on 1033 AN patients vs. 3733 controls confirmed that a common variant of *OPRD1* gene is indeed associated with AN (Wang et al., 2011), although not in the more recent GWAS which was performed on a larger sample (Boraska et al., 2014).

The level of opioids in the nucleus accumbens (NA) critically regulates the release of dopamine. A positron emission tomography performed on 10 recovered AN patients and 12 controls reported an increased binding of dopamine D2/D3 receptors in the anterior ventral striatum, which contains the NA (Frank et al., 2005). This difference could be due to either an increase of density and/or affinity of the D2/D3 receptors in the NA, or a decrease of dopamine level in AN. It is thus possible that AN patients reduce their food intake because of abnormalities of dopamine dysfunction, i.e., of the reward brain circuit, transmitting an anxiogenic signal instead of an expected hedonic one. Genetic variants of the *DRD2* gene, including -141C Ins/Del previously shown to modify transcription level, were associated with AN (Bergen et al., 2005). Furthermore, a significant higher percentage of methylated promoter of *DAT1* and *DRD2* genes was observed in AN patients compared to controls, and such methylation were associated with a increased expression of the *DAT1* gene and a decreased expression of the *DRD2* gene (Frieling et al., 2010). Thus, potential genetic and epigenetic dysregulations of the dopamine reward circuit in patients reinforce its role in the pathophysiology of AN. In addition, the receptor of the hunger hormone ghrelin is able to form heterodimers with DRD2 in hypothalamic neurons, potentially promoting anorexigenic behavior (Kern et al., 2012).

**Figure 1:**
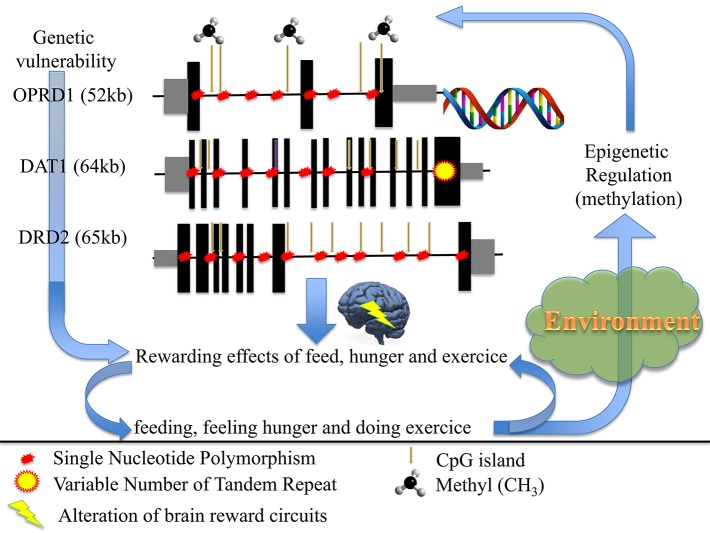
**How genetic and epigenetic factors could influence the risk and/or the maintenance of anorexic behaviors (driving for further thinness while underweight)**.

To conclude (Figure 1), we propose that anorexia nervosa results from dysregulation(s) of the balance between input (feeding/hunger) and output (excessive exercise), also at the genetic and/or epigenetic levels, of the dopamine genes involved in (1) the reward circuitry, located in the ventral striatum, and (2) the food regulatory mechanism, located in the hypothalamus, to alter these processes and confer starvation dependence.

## Model 2: anorexia nervosa is a ghrelin-specific resistance?

AN patients exhibit changes in the release of hormones involved in energy metabolism and regulation of feeding behavior (Germain et al., 2007; Hasan and Hasan, 2011). In particular plasma levels of ghrelin, an orexigenic hormone mostly released from the stomach (Cummings et al., 2001), are increased (Germain et al., 2009, 2010). Such an increase seems paradoxical in light of the restrained eating adopted by these patients, but may be adaptive by a feedback mechanism due to the lack of nutrients. Several groups have proposed the concept of ghrelino- resistance that reflects the inability of increased ghrelin to induce appetite in AN patients, thereby creating a metabolic vicious circle maintained by their food restriction behavior. In this context, the ghrelin system should be considered as a valuable therapeutic target in eating disorders.

### Ghrelin is derived from a unique prohormone coding various peptides involved in feeding-oriented behaviors

**Figure 2:**
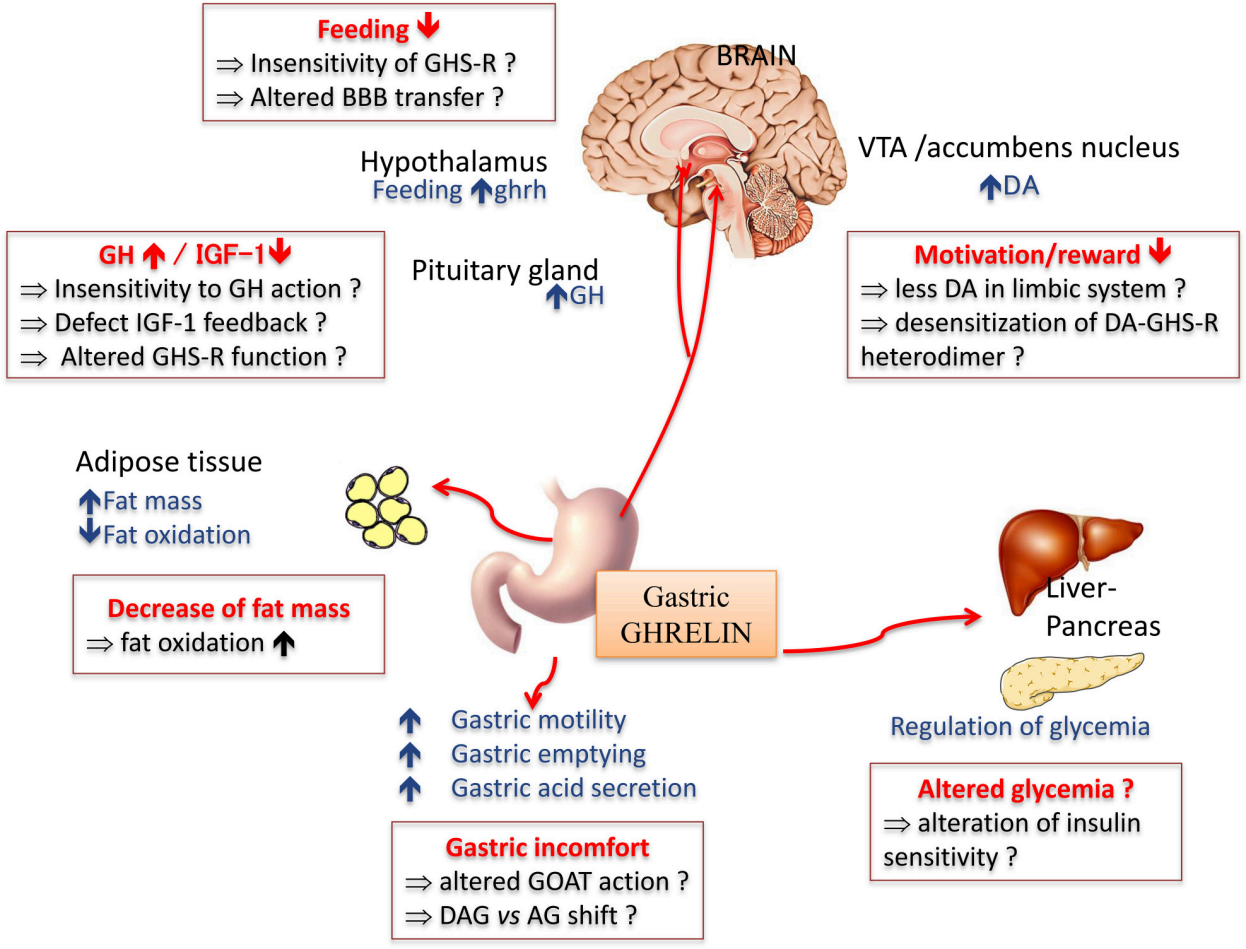
**Main physiological effects of the orexigenic hormone ghrelin**. In anorexia nervosa, some of the symptoms classically described might be due to a resistance or insensitivity to ghrelin effect (in red squares). AG, acyl ghreline; BBB, blood brain barrier; DA, dopamine; OAG, deacyl ghrelin; GH, growth hormone; GHS-R, ghrelin receptor; GOAT, ghrelin O -acyltransferase; IGF-1, insulin growth factor 1; VTA, ventral tegmental area.

Amongst peripheral factors sensitive to nutritional, hedonic and emotional signals, preproghrelin is a unique prohormone encoding several peptides with structural and functional heterogeneity (Hassouna et al., 2010). Ghrelin is synthetized from the stomach (Kojima et al., 1999; Tomasetto et al., 2000) and was initially identified as an endogenous ligand for the growth hormone (GH) secretagogue receptor (GHS-R1a). In addition to its primary effect as a GH secretagogue (Tolle et al., 2001), ghrelin exerts pleiotropic effects both peripherally and centrally (Figure 2), including the modulation of the dopaminergic reward system (for review see Méquinion et al., 2013). Other naturally occurring variants of ghrelin are desacyl ghrelin (DAG), which is the most abundant form in plasma and accounts for 80–90% of total ghrelin (Kojima and Kangawa, 2005) and obestatin, another bioactive peptide initially described for its anorectic actions in rodents (Zhang et al., 2005).

### Clinical evidence of ghrelino-resistance

Plasma acyl ghrelin (AG) levels are elevated in AN with a pure restrictive type (AN-R) (Germain et al., 2009, 2010). Investigations of pharmacological effects of ghrelin in patients with AN are not conclusive as yet. Indeed, intravenous infusion of ghrelin during 5 h induced no clear changes in food intake, but increased sleepiness (Miljic et al., 2006). A sensation of hunger was shown in six of nine patients with AN after a bolus intravenous injection of 1 mg/kg ghrelin (Broglio et al., 2004) similarly to normal subjects (five of seven responders). Another study demonstrated that administration of ghrelin (3 mg/kg) twice a day decreased gastrointestinal symptoms and increased sensations of hunger and daily energy intake (12–36%) on five AN patients (Hotta et al., 2009). In these pilot studies, the true effect of ghrelin remains difficult to assess due to the lack of proper controls, the small number of treated patients, difficulties to properly evaluate the real motivation of patients to eat and the route and frequencies of ghrelin and/or agonists administration (Ueno et al., 2010).

### If ghrelino-resistance exists, where and how does it occur?

The inability of AN-R patients to respond to elevated ghrelin levels could be due to: a reduced ability to be transported to central targets within the hypothalamus (Schaeffer et al., 2013); a reduced GHS-R sensitivity or function; a dysregulation of hypothalamic NPY/AgRP and POMC neurons, direct or indirect targets of ghrelin effects, exerting opposing actions on food intake (Denis et al., 2014); an alteration in the ghrelin signaling on dopaminergic neurons, in the ventral tegmental area (VTA) (Abizaid et al., 2006), a structure involved in the reward/locomotion behavior, and hypothalamus to modulate appetite, respectively (Jiang et al., 2006; Kern et al., 2012; Sharpe et al., 2016).

### Ghrelino-resistance: antagonism by other ghrelin-derived peptides?

The hypothesis of a ghrelino-resistance in AN-R requires further evidence that (1) endogenous ghrelin is important in stimulating appetite and (2) other ghrelin-derived peptides might be modulators of AG actions in animal models and AN patients.

In mice with total ghrelin deficiency or selective AG deficiency, food intake is not reduced (Sun et al., 2003, 2004; Zigman et al., 2005; Pfluger et al., 2008; Zhao et al., 2010). This does not seem to support a key role of AG in homeostatic eating. However, suppressed intake of rewarding food in a free choice food paradigm, lack of cue-potentiated feeding and suppressed motivation for food in an operant responding model rather support a role of the endogenous peptide(s) in hedonic eating (Uchida et al., 2013).

DAG may be an important modulator of appetite by counteracting AG actions as demonstrated after central or peripheral injections (Asakawa et al., 2005; Inhoff et al., 2008). Interestingly, transgenic mice overexpressing DAG display significantly reduced body weight, reduced food intake and body fat mass and moderately decreased linear growth compared to non-transgenice mice (Asakawa et al., 2005). In addition, DAG exerts several biological actions, independent of the GHS-R pathway, including regulation of glucose homeostasis and fat metabolism (Delhanty et al., 2012, 2013). Although obestatin was initially reported to decrease appetite and weight gain thus opposing ghrelin's effects by acting on an orphan receptor, GPR39 (Zhang et al., 2005), controversies rapidly arose concerning the anorexigenic actions of this peptide through GPR39 (Lauwers et al., 2006; Chartrel et al., 2007; Holst et al., 2007; Zizzari et al., 2007; Hassouna et al., 2010, 2012). In AN-R patients, obestatin, and the ghrelin/obestatin ratio were found decreased whereas this ratio was not altered in constitutionally thin women (Germain et al., 2009, 2010). Such changes may participate in the eating restriction despite hyperghrelinemia observed in these patients.

### Other possible mechanisms to explain resistance to feeding

Ghrelin might also impact non-homeostatic systems that involve more particularly the meso-cortico-limbic system. Skibicka et al. (2013) demonstrated that the significant increase for food motivation/reward behavior observed after a ghrelin injection in the VTA is abolished by a pretreatment with D1-like or D2 receptor antagonist, injected in the nucleus accumbens, the main target of VTA dopaminergic neurons. This brain structure is also involved in the integration of messages from the prefrontal inhibitory circuitry. Indeed, prefrontal cortex is strongly activated in AN patient following presentation of food images as observed in a fMRI study (Zhu et al., 2012), and alterations in the serotoninergic/dopaminergic signaling are also described in these patients (Bailer et al., 2007, 2013). In AN patients, an altered impact of ghrelin on the reward systems might modify the integration of informations related to emotional process associated with food. Due to the paucity of data and the lack of consistent clinical information, the use of animal models to analyze more finely the mechanisms of ghrelin action at different levels of the brain system appears essential.

### From clinics to bench: what can we learn from animal models?

A complete animal model of AN does not exist due in part to the neuropsychiatric aspects of the disease (Méquinion et al., 2015b). Such an ideal model of AN would include adolescent onset of the disease, predominance in females, high activity, chronic stress, decreased food intake, and body weight. Moreover, AN is a chronic disease, and most of the murine models described so far maintain a protocol on a short period (few days). Neverheless, some aspects like food restriction associated with voluntary physical activity or chronic stress can be reproduced in mice models. The model of separation with restricted access to food (separation-based anorexia, SBA), first developed in mice by van Leeuwen et al. (1997), induces a chronic stress that results in an increase of energy expenditure without hyperactivity. Recent data demonstrated that, when this protocol is maintained on the long-term, it induced AN like alterations such as a decrease in bone mineral density (osteoporosis) associated with a dysregulation of leptin signaling (Zgheib et al., 2014). The model of hyperactivity associated with food restriction (activity-based anorexia, ABA), first developed in rats (Hall et al., 1953; Routtenberg and Kuznesof, 1967) and subsequently adapted to mice (Gelegen et al., 2007) is considered as a good animal model of AN as it mimics important features of the disease (Routtenberg and Kuznesof, 1967; de Rijke et al., 2005). The ABA model also emphasizes the involvement of the ghrelin/GHS-R pathway in food anticipatory activities, a behavior also described in AN-R patients (Blum et al., 2009; Scheurink et al., 2010; Verhagen et al., 2011). On a modified long-term ABA model, where food was quantitatively restricted (50% during 10 weeks), symptoms classically observed in AN patients (body weight loss, endocrine changes, oestral cycle loss…) as well as alterations in the integration of the ghrelin message at the hypothalamic levels were observed (Méquinion et al., 2015a).

### Conclusion: is anorexia nervosa a ghrelino-resistance or an adaptative metabolic response to denutrition?

In human pathology, the question remains whether ghrelin resistance (or decreased sensitivity) is only limited to the balance between patients' hunger and satiety and motivation to the rewarding aspect of food. Indeed, such hyperghrelinemia can also serve as an adaptive neuroendocrine metabolic response to the disease since ghrelin appears required for the maintenance of blood glucose homeostasis during severe calorie restriction in mice (Zhao et al., 2010). Thus, up-regulation in ghrelin synthesis may be needed to maintain survival in the restrictive type of AN. However, ghrelin is differentially regulated according to the anorexic subtype as plasma levels are found unchanged in binge-purging anorexic patients, suggesting that both subtypes engage in differential adaptive process to starvation and/or a difference in the perception of hunger and the motivation to eat. Extrapolation from these fundamental observations to the human pathology should be carefully interpreted and will require further exploration and validation of experimental models. Animal models, despite their weakness on some aspects of the disease might permit to assess long term peripheral and/or central alterations due to chronic and drastic food restriction associated or not with activity or chronic stress. Anxiety and hyperactivity behaviors have a significant role in the pathogenesis and progression of the disease. One of the major advantages of using mice as an animal model is that many well-described inbred lines are available to assess the part of genetic or environmental component of anorexia. These animal models will be useful to precisely determine the mechanisms involved in the impaired integration of the ghrelin signal.

## Model 3: anorexia nervosa (AN) is a chronic stimulation of the reward system by orexigenic neuropeptides of the lateral hypothalamic area

The hypothalamus plays a pivotal role in behavioral and emotional reactions and contains the major center that regulates feeding behavior. All patients are unable to adapt their feeding behavior to energy demand and costs, raising the possibility of a dysfunction of the orexigenic and/or anorexigenic neuropeptides regulating appetite within the hypothalamus. Besides, recent evidence support the view that AN might be a pathology of abnormally high reward process for starvation or purging behaviors. For instance, patients recovered from AN still show an increased neural response to both pleasant and aversive food stimuli in brain circuitry mediating reward (Cowdrey et al., 2011). Dopamine is the neurotransmitter of the reward system and several lines of evidence suggest the occurrence of a dysfunctional dopamine-striatal system in AN that might explain the lack of pleasure associated with food intake and the general anhedonia of anorectic patients (Kaye et al., 2009). Supporting this notion, amphetamine-induced dopamine release increases anxiety in individuals recovered from AN (Bailer et al., 2012) and that could explain why food-related dopamine release generates anxiety in AN patients whereas feeding is pleasurable in healthy subjects. Interestingly, the lateral hypothalamic area (LHA), that contains the orexigenic neurons expressing orexins, melanin-concentrating hormone (MCH), and 26RFa, sends projections to the brain reward circuitry (Aston-Jones et al., 2010), suggesting that dysfunction of orexigenic hypothalamic neuropeptides may alter the dopamine reward system in AN.

**Figure 3:**
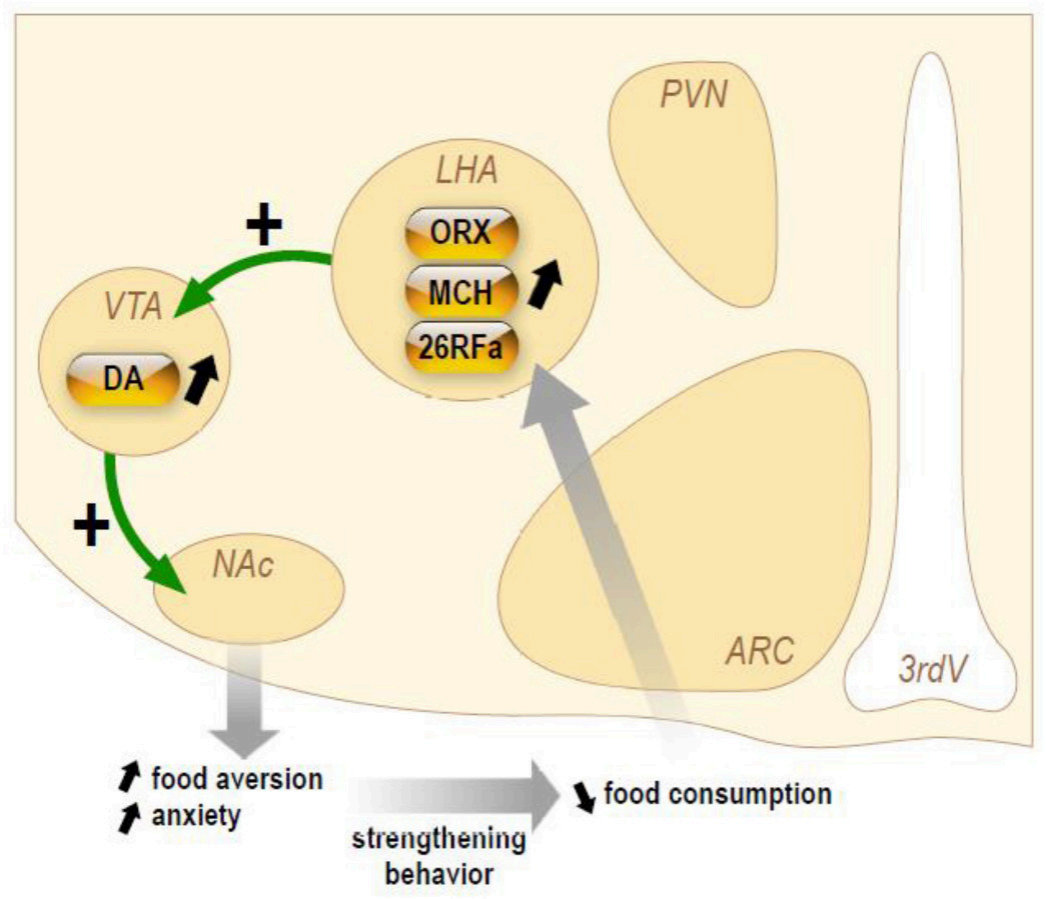
**Schematic representation of the hypothetical chronic stimulation of orexigenic neuropeptides on the reward circuitry in anorexia nervosa**. Briefly, decrease of food ingestion induces a stimulation of the neuronal activity of the lateral hypothalamic area (LHA) that will release the orexigenic neuropeptides orexins (ORX), melanin-concentrating hormone (MCH), and 26RFain the ventral tegmental area (VTA). This results in an increase of dopamine (DA) release in the accumbens nucleus (NAc). In anorectic patients, this stimulation of the reward system results in food aversion associated with enhanced anxiety that will, in turn, reinforce the decrease in food intake.

Herein, we will briefly present (Figure 3) the current knowledge on three orexigenic neuropeptides produced by neurons of the LHA in AN, i.e., the orexins, MCH, and 26RFa.

### Orexins

Orexins are produced by <80,000 neurons exclusively located in the LHA (Sakurai et al., 1998). Orexin A (OxA) and orexin B (OxB) are two neuropeptides that share 46% identity and are in tandem on the same precursor (Sakurai et al., 1998). Orexins are the endogenous ligands of two G-protein coupled receptors termed OxR1 and OxR2 (Sakurai et al., 1998). Central administration of OxA or OxB stimulates food consumption (Sakurai et al., 1998). Prepro-orexin mRNA is up-regulated following fasting, and conversely down-regulated in *ob/ob* and *db/db* obese mice (Sakurai et al., 1998; Yamamoto et al., 1999), whereas orexin deficient mice show robust hypophagia (Hara et al., 2001). Furthermore, systemic administration of the OxR1 antagonist SB-334867 reduces feeding by selectively enhancing the behavioral satiety (Ishii et al., 2004).

Two distinct studies have evaluated the evolution of plasma OxA levels in patients with AN during 2, 3, or 6 months of realimentation (Bronsky et al., 2011; Janas-Kozik et al., 2011). The data obtained are conflicting as the first study (Bronsky et al., 2011) reports a significant increase of fasting plasma OxA concentration in untreated AN patients whereas the second one describes a decrease of circulating OxA in AN subjects before refeeding (Janas-Kozik et al., 2011). However, the two studies observe a progressive decrease of circulating OxA during realimentation, rather suggesting that the orexin system is up-regulated in AN.

Accumulating data indicate that orexins are involved in reward-based feeding. As a matter of fact, activation of orexin neurons in the LHA is strongly linked to preferences for cues associated with drug and food reward (Harris et al., 2005). Consistent with this, orexin injected into the VTA, that contains the dopamine neurons, increases dopamine release in the accumbens nucleus (NAc), which provides enjoyment and reinforcement to motivate repeated activity, and stimulates intake of a rewarding high fat diet (Zheng et al., 2007). Conversely, OXR1 antagonism in VTA attenuates high-fat feeding in sated rat (for review, Cason et al., 2010). Additionally, central administration of orexin increases self-administration for sweet, an effect that is blocked by OXR1 antagonists, suggesting that orexins promote the reinforcing properties of at least some types of food (Cason et al., 2010).

### MCH

MCH is a cyclic peptide predominantly expressed in the LHA. MCH induces food intake after central injections (Qu et al., 1996), and its gene expression is modulated by fasting and in hypoleptinemic *ob/ob* mice (Qu et al., 1996). Two G protein-coupled (GPCR) MCH receptors (MCH-R1 and MCH-R2) are present in human, of which only MCH-R1 appears functional in rodents (Forray, 2003). Several genetically-modified mice that overexpress or lack MCH (Shimada et al., 1998) or MCH-R1 (Marsh et al., 2002) genes were generated and displayed marked alterations in the regulation of appetite and/or energy balance. At present, the MCH system has never been investigated in AN. However, it is interesting to note that the orexigenic effect of MCH is largely dependent upon its action in the NAc of the reward system. Indeed, injections of MCH and MCH antagonists into the NAc are orexigenic and anorexigenic respectively (Georgescu et al., 2005). MCH injections in this area decrease neuronal firing of spiny neurons and increase hedonic value of sweet foods (Lopez et al., 2011). This suggest that MCH signaling pathways play a complex but important role in feeding-reward control and could provide important information about mechanisms underlying the development of AN.

### 26RFa

26RFa is a 26-residue RFamide peptide characterized in various vertebrate species including Man (Chartrel et al., 2003, 2011). 26RFa has been identified as the cognate ligand of the human orphan G protein-coupled receptor, GPR103 (Takayasu et al., 2006; Chartrel et al., 2011). Neuroanatomical observations revealed that 26RFa- and GPR103-expressing neurons are primarily localized in hypothalamic nuclei involved in the control of feeding behavior (Chartrel et al., 2003; Takayasu et al., 2006; Bruzzone et al., 2007). 26RFa-containing neurons are exclusively located in the LHA and the ventromedial hypothalamic nucleus (Chartrel et al., 2003). Central administration of 26RFa stimulates food intake and causes obesity in rodents (Chartrel et al., 2003; Moriya et al., 2006; Takayasu et al., 2006). In addition, 26RFa mRNA is up-regulated in the hypothalamus of obese *ob/ob* and *db/db* mice (Takayasu et al., 2006) indicating that the expression of the 26RFa gene is modulated in condition of energy imbalance.

Circadian profile of plasma 26RFa was investigated in AN patients. The data showed that 26RFa levels are significantly increased all over the day in patients with restrictive AN as compared to healthy subjects (Galusca et al., 2012).

Detailed mapping of GPR103 mRNA in the brain revealed that the 26RFa receptor is expressed in the VTA, the amygdala, and the NAc (Bruzzone et al., 2007), suggesting that, in as much the same way as orexins and MCH, 26RFa may be involved in reward-based food intake.

### Conclusion

Orexigenic neuropeptides including ghrelin, orexins and 26RFa are up-regulated in AN and it is thought that this orexigenic profile reflects an adaptive mechanism of the organism to promote food intake and thus to counteract undernutrition. However, this adaptive mechanism is ineffective in increasing food consumption leading to the concept of a global resistance of AN patients to orexigenic signals. Here we present an alternate hypothesis. We speculate that a chronic increase of the activity of LHA orexigenic neurons expressing orexins, MCH, or 26RFa could reinforce dopamine-induced anxiety in the reward system of AN patients and thus the aversion to ingest food.

## Model 4: gut microbiota is a central factor in anorexia nervosa

Anorexia nervosa (AN), the most frequent and the most serious eating disorder, is often associated with severe proteino-energetic malnutrition (PEM). Marasmus, the adaptative form of semi-prolonged fasting, is the predominant form of malnutrition associated with AN. However, the kwashiorkor type is present in some cases, and is characterized by a constellation of features including peripheral edema, hypoalbuminemia, fatty liver, skin and hair lesions, apathy, high a relative immuno-depression with high risk of infections. It is associated with a lot of deleterious secondary complications and a high rate of morbidity/mortality. Some patients have a mixed picture of marasmic-kwashiokor malnutrition.

In the last decades, the role of gut microbiota in different diseases was demonstrated thanks to the development of new methods. The most accurate ones are metagenomics which aims to study all genetic content of prokaryote cells by high throughput sequencing.

With such new tools, the role of gut microbiome was defined on nutriments absorption and energy regulation (harvest, storage, and expenditure of energy). It is now established that the gut microbiome contributes to the risk and pathogenesis of malnutrition through nutrient metabolism and immune function (Krajmalnik-Brown et al., 2012). A clinical trial, comparing 16s rRNA PCR fecal samples of seven malnourished children with seven healthy ones, showed that the former have a significantly lower number of operational taxonomic units in their gut than healthy subjects (310 vs. 546) (Monira et al., 2011). A metagenomic comparison of gut microbiota, studying 9 well-nourished twin pairs to 13 twin pairs discordant for kwashiorkor, showed a significant difference on gut microbiota profile. Indeed malnourished twins (kwashiorkor) had a considerably less differentiated and immature gut microbiome than healthy or marasmic children (Smith et al., 2013). When stool samples from kwashiorkor-malnourished children were transplanted to gnotobiotis mice, these animals developed weight loss and severe malnutrition (Smith et al., 2013). In a recently randomized controlled clinical trial in 2767 Malawian malnourished children, a 1-week oral antibiotic (which modulates gut microbiota) treatment improved the nutritional status of malnourished children and decreased mortality (Trehan et al., 2013).

Modifications of the gut microbiota have also been described in AN patients. Armougom et al. (2009) measured bacterial divisions of *Bacteriodetes, Firmicutes, Lactobacillus*, and *Methanobrevibacter smithii*. They compared the feces of 20 obeses subjects, 9 patients with AN and 20 normal weight healthy controls. AN patients were moderately undernourished, with an average BMI at 16. All AN patients had an increase of *M. smithii* (Armougom et al., 2009). This Archeon, which comprises up to 10% of all anaerobes in the colon of healthy adults, plays a central role in bacterial digestion of polysaccharides. It recycles hydrogen in methane, allowing for an increase in the transformation of nutrients into calories and thereby increasing energy efficiency (Samuel et al., 2007). It is possible that chronic constipation, which is common in AN patients, could also be present before weight loss and causes the increase of *M smithii* in their gut (Kim et al., 2012). In addition, by using electrophoretic profile of the 16S RNA microbiota, a deviation of microbiota was found in 8 AN undernourished patients (average BMI at 12) in comparison to nine healthy subjects (Hanachi et al., 2013).

All these data show that there are modifications in gut microbiota of AN patients, not necessarily severely malnourished. These changes include an increase of methanogen archea directly involved in energy efficiency. Indeed the increase of *M. smithii* can lead to the optimisation of food transformation of nutrients into calories. This increase of *M. smithii* could have preceded undernutrition and could play a central role in the outbreak of anorexia nervosa. In order to explore this pathway the gut microbiota of patients with AN should be assessed before the beginning of weight loss, during nutritional therapy and after recovery of weight loss.

**Figure 4:**
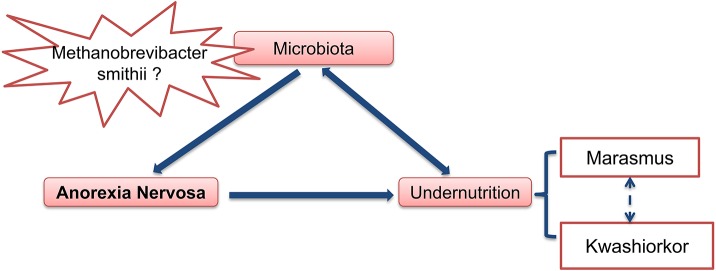
**Gut microbiota as an important contributing factor in Anorexia Nervosa**.

In summary (Figure 4), at least two hypotheses can be proposed concerning the role of gut microbiota in anorexia nervosa: (1) some pre-existing changes in gut microbiota induce and/or participate to food restriction in these patients and chronic constipation could induce and/or facilitate this change in gut microbiota. (2) The gut microbiota determines for each individual patient the type of PEM, i.e., marasmus type vs. kwashiorkor. In such case, manipulation of gut microbiota during AN (with antibiotics for example), might improve the issue of malnutrition during nutritional therapy.

## Model 5: anorexia nervosa as a dysimmune disorder of neuropeptide signaling

**Figure 5:**
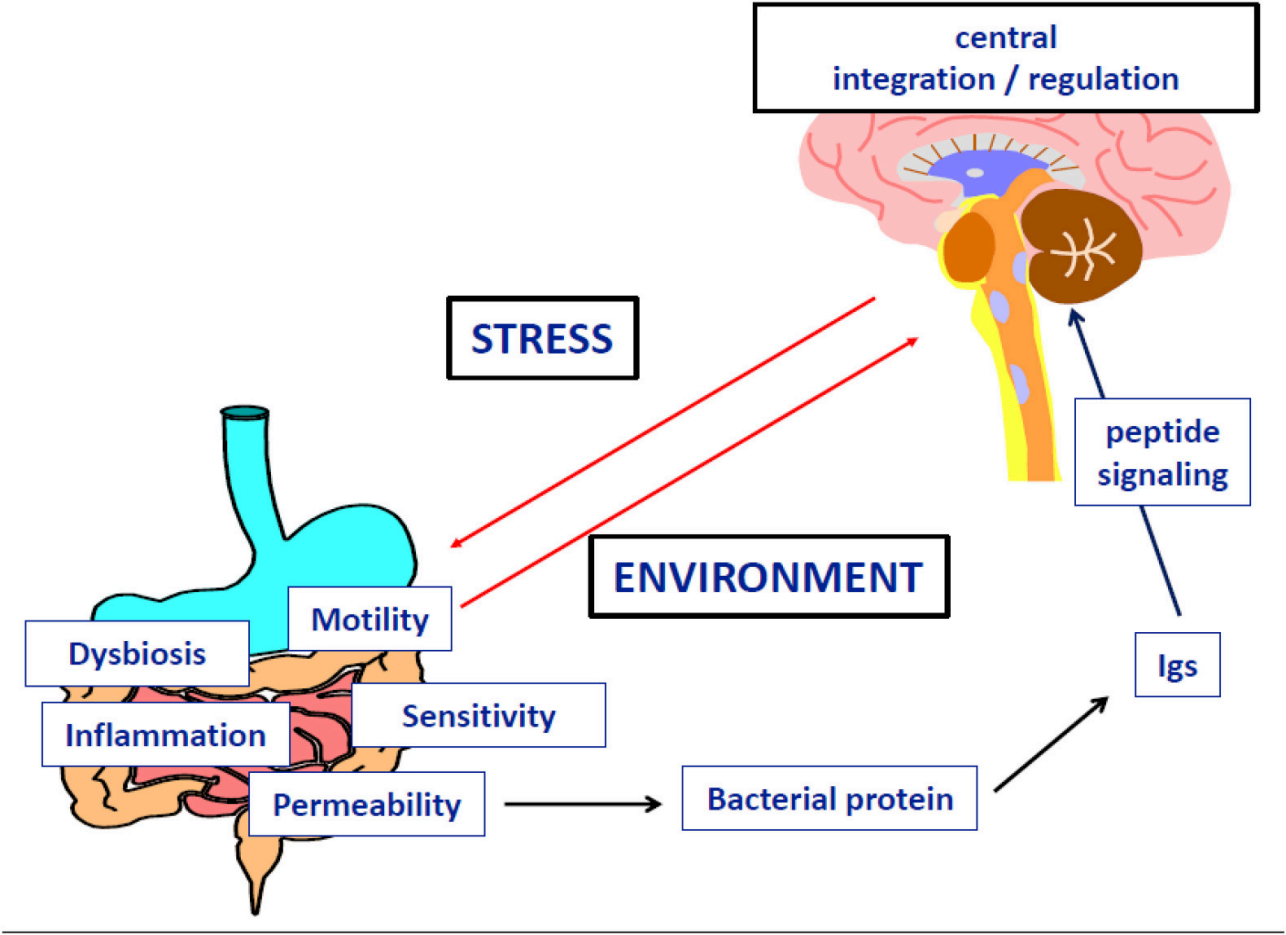
**Implication of microbial protein and specific Immunoglobulins in the dysfunction of neuropeptide signaling during anorexia nervosa**.

We review here recent evidence from experimental and clinical studies explaining how stressors could result in the dysregulation of eating behavior by the means of specific immunoglobulins (Igs) altering neuropeptide signaling (Figure 5). We also discuss a key role of intestinal bacteria and gut barrier dysfunction in this dysregulation. Although anorexia nervosa (AN) is classified as a psychiatric disease, the clinical observations that it involves multiple organ dysfunction, in addition to the core behavioral and psychiatric symptoms, underlines the need for an integrative pathophysiological approach. Anorexia is generally considered as the driving mechanism leading to malnutrition and its multiple organ consequences, leading in turn, to the perpetuation of anorexia and psychiatric comorbidity in a vicious circle. However, this classical approach does not clarify how anorexia and related symptoms may occur in so far healthy adolescents or adults. It is widely accepted that the risk of developing AN is markedly increased by different types of stressors: physical (such as puberty, trauma, abuse, or infections) and/or mental stress, including for example intellectual or physical exhaustion or repeated dieting (Hilbert et al., 2014). Familial history of stress events or stress-related disorders is another risk factor of AN, suggesting that genetic or epigenetic factors, so far poorly identified, may contribute to the maladjusted response to stress in patients developing AN (Trace et al., 2013). Finally, over the past 10–20 years, rapidly growing knowledge on the physiology of eating behavior identified neuropeptides and peptide hormones as major signaling molecules, in addition to monoamines, to regulate food intake, satiety, as well pleasure and other behaviors and functions that may be altered during AN, e.g., sleep, anxiety, digestive motility, and sensitivity, endocrine functions and bone metabolism (Berthoud, 2011). This led to the concept of neuropeptide signaling dysfunction in AN (Inui, 2001), but an approach based only on peptide concentrations measurements and classical hormone-receptor interaction provided conflicting or paradoxical findings (Prince et al., 2009), e.g., that food intake remains low in anorectic patients despite increased orexigenic ghrelin (Otto et al., 2001) and decreased anorexigenic leptin (Hebebrand et al., 1995) levels.

We have performed a series of clinical and experimental studies indicating that neurobiological mechanisms of eating disorders and obesity may involve Igs reactive with peptide hormones regulating food intake and emotion, therefore also called peptide auto-antibodies (Fetissov et al., 2002). The initial finding was the identification of Igs in the serum of patients with AN or bulimia nervosa (BN) binding to α-melanocyte-stimulating hormone (α-MSH) in hypothalamic neurons (Fetissov et al., 2002). The clinical relevance of these Igs was further suggested by the correlation of their plasma levels and the Eating Disorder Inventory-2 (EDI-2) scores in AN and BN patients (Fetissov et al., 2005). To elucidate the potential origin of these Igs, an *in silico* search was performed, based on the concept of molecular mimicry, with the hypothesis that some microorganisms present in the environment, especially in gut microflora, may present sequence homology with neuropeptides. This hypothesis was confirmed for several common microorganisms expressing in several proteins identical five consecutive amino acids with the main peptides involved in appetite, satiety, or emotion (e.g., α-MSH, ghrelin, leptin, orexin; Fetissov et al., 2008). In addition, both IgG and IgA classes of Igs reactive with peptides hormones were present in healthy subjects, further supporting a luminal source of antigens as well as physiological role of these Igs in the modulation of neuropeptide signaling. In support of this, other authors have shown the stimulatory effects of gut microbiota on all Igs classes in germ-free mice (Hansson et al., 2011).

With respect to AN pathophysiology, several of our studies focused on anti-α-MSH Igs, since this 13 amino acid peptide is a major regulator of energy balance, increasing satiety and energy expenditure via the activation of the melanocortin receptor type 4 (MC4-R), and is closely related to the hypothalamic response to stress (Cone, 2006). With this in mind, a study was undertaken in rodents showing the impact of chronic stressors on the levels and binding properties of anti-α-MSH Igs, related to food intake and anxiety-like behavior (Sinno et al., 2009). Furthermore, in a rat model of severe gut inflammation, inducing transient anorexia and major increase of intestinal permeability, deficient body weight gain persisted even after the resolution of intestinal inflammation and was associated with an increased level of anti-α-MSH Igs (Coquerel et al., 2012). We also adapted an animal model of AN called Activity-Based-Anorexia (ABA), combining a progressive reduction of time access to food coupled with running wheel to mimic the hyperactivity of AN patients. We found that ABA mice were characterized by an increased colonic permeability which is favorable for the passage of antigens from gut bacteria to the host (Jésus et al., 2014).

To explore the implication of bacterial proteins from gut microflora in the occurrence of peptide-reactive Igs, we used a combined proteomic and immunological approach to identify α-MSH antigen-mimetic proteins in Escherichia *coli*. The commensal *E. coli* K12 was used because several *E. coli* proteins display five amino acids sequence homology with α-MSH (Fetissov et al., 2008). We identified ClpB, a heat-shock chaperone protein, as a conformational antigen mimetic of α-MSH (Tennoune et al., 2014). We showed that immunization of mice with ClpB induced the production of anti-ClpB Ig cross-reacting with α-MSH accompanied by altered food intake, body weight, anxiety and MC4-R signaling. Furthermore, mice receiving wild-type *E. coli* via intragastric gavage, decreased food intake and body weight gain and developed anti-ClpB- antibodies cross-reactive with α-MSH. In contrast, in mice receiving ClpB-deficient *E. coli*, food intake and Igs levels remained unaltered. Finally, plasma levels of anti-ClpB IgG cross-reactive with α-MSH were increased in patients with eating disorders, and several significant correlations were found between EDI-2 scores and anti-ClpB IgG and IgM levels in AN patients (Tennoune et al., 2014), similar to previous findings with anti-α-MSH reactive Igs (Fetissov et al., 2005). These data revealed that α-MSH-reactive Igs in humans and animals are generated mainly in response to bacterial ClpB protein produced by some gut bacteria including *E. coli*. It is of relevance to mention that the ClpB protein expression in bacteria is stimulated by various stressors preventing protein aggregation (Winkler et al., 2012), suggesting that ClpB could also be activated by host-induced starvation during a restrictive diet.

Altogether, these studies indicate that different stressors, including food restriction, via increased intestinal permeability, may favor bacterial protein translocation from the gut resulting in increased production of neuropeptide-reactive Igs. More specifically, bacterial protein ClpB may act as an anorexigenic α-MSH mimetic protein directly and indirectly via triggering production of Igs cross-reactive with α-MSH, and hence, altering the effects of this endogenous peptide hormone on satiety and anxiety. This may involve a modulatory role of Igs on α-MSH-induced activation of the MC4-R (Lucas et al., 2014), although the exact mechanisms needs further investigation. Other groups have suggested that gut microbiota, especially *E. coli*, may influence the stress axis (Dinan and Cryan, 2012), while *E. coli* content was inversely related to the body mass index, including AN patients (Million et al., 2013). Studies on gut microbiota involvement in brain functions are discussed elsewhere (Cryan and Dinan, 2012); our data support a role of bacterial protein mimetics of peptide hormones in the gut-brain axis communication and validate the utility of proteomic approach as an efficient tool to detect such proteins.

In addition to the putative key role of α-MSH-mimetic protein and α-MSH-reactive Igs in AN pathophysiology, it is probable that other peptidergic systems involved in regulation of feeding and emotion can be altered via a similar mechanism involving the gut-brain communications. Accordingly, the levels and properties of Igs reactive with ghrelin were altered in AN patients (Terashi et al., 2011), which might account with the previously proposed ≪ ghrelin resistance ≫ in these patients (Otto et al., 2001). In an opposite way, the affinity kinetics of ghrelin-reactive Igs in obese patients explained its increased orexigenic effects, through its reduced degradation (Takagi et al., 24). Whether ghrelin-reactive Igs in AN patients may alter activation of the ghrelin receptor remains to be explored. Orexin-reactive Igs have been detected in narcolepsy (Deloumeau et al., 2010), which raises question about their possible implication in sleep disturbances during AN. It is worth underlining that several neuropeptides controlling food intake and anxiety, are also involved in gastrointestinal motor and sensitive regulation (e.g., ghrelin, CCK), which may explain the high prevalence of gastrointestinal symptoms during AN (Déchelotte et al., 2007).

In conclusion, we propose an integral pathophysiological scenario that fits to the natural history of AN with the following steps: (1) enhanced vulnerability to stress (genetic, epigenetic, or environmental factors); (2) major stressing events activating the stress-axis, increased intestinal permeability, and increased virulence of the microbiota; (3) bacterial proteins (e.g., ClpB) challenge the immune response and due to molecular mimicry, cause increased production of Igs cross-reactive with neuropeptides (e.g., α-MSH); (4) this results in altered food intake, anxiety, gastrointestinal discomfort and other consequences of altered central and peripheral melanocortin signaling; (5) global malnutrition and some specific macro- and micro-nutrient deficiencies contribute to the perpetuation of gut barrier and immune dysfunction as well as behavioral symptoms. This scheme opens potential therapeutic perspectives in AN, involving a multimodal approach including behavioral and/or pharmacological reduction of stress, restoration of gut barrier function, correction of microbiota imbalance, and specific targeting of bacterial proteins and/or related Igs.

## Model 6: anorexia nervosa takes root in the pondero-nutritional-eating basements during childhood's premorbid period

**Figure 6:**
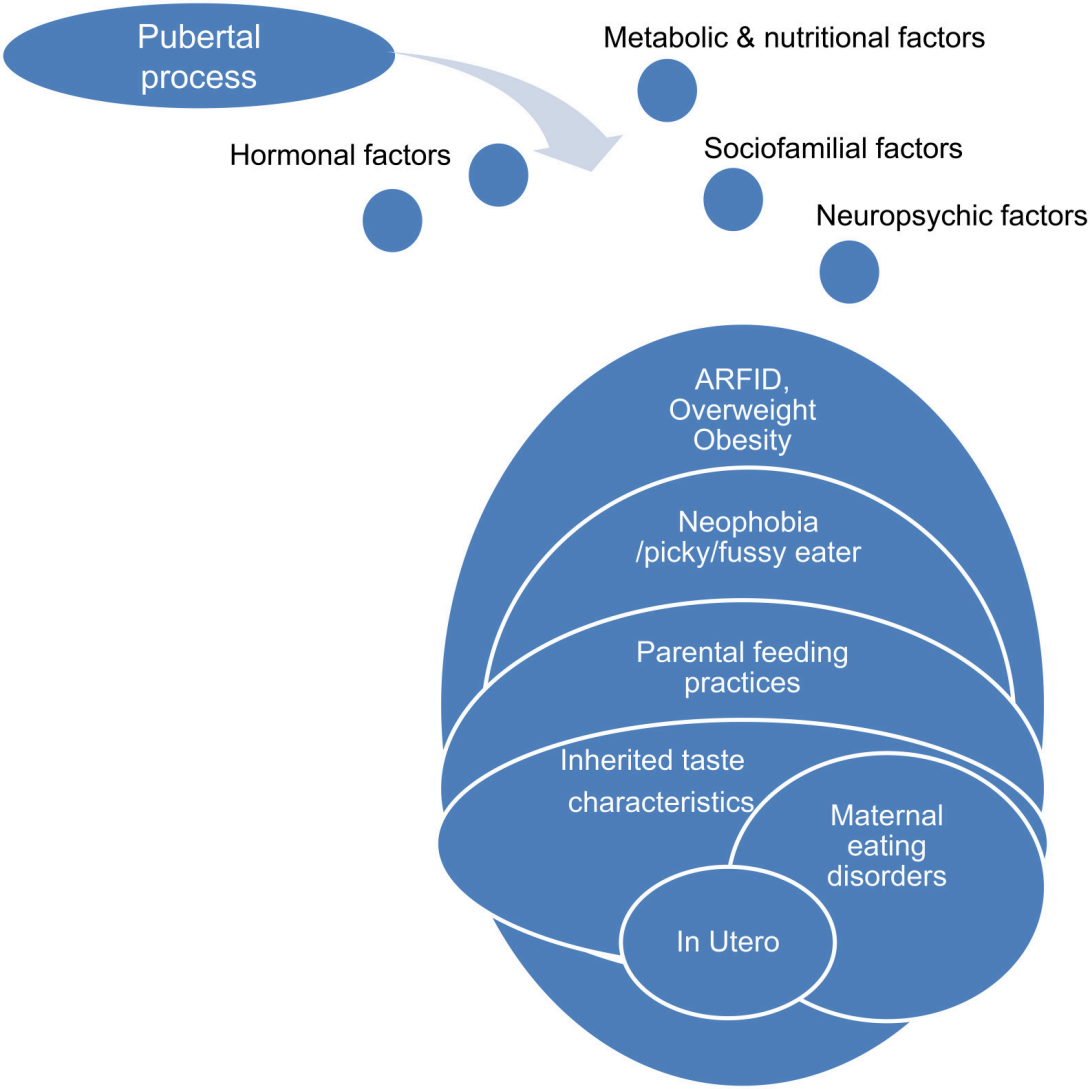
**Pubertal process and pondero-nutritional-eating basements: the mismatching team**.

Physiological puberty process, partially genetically determined, is associated with strong physical, psycho-affective, hormonal, metabolic, and feeding behaviors variations. One possible hypothesis is that anorexia nervosa during adolescence is a kind of mismatch of this physiological process, permitted by underlying and premorbid vulnerability factors concerning the ≪ pondero-nutritional-feeding ≫ basements (Figure 6). Susceptibility factors existing during the premorbid period may concern development of taste, food choice, eating behaviors. The pubertal process stimuli interfering with these vulnerability factors could promote a failure of the ≪ pondero-nutritional-feeding ≫ system homeostasis resulting in later anorexia nervosa. The vulnerable system is a kind of trigger zone account for the symptom choice during difficult pubertal process explaining how initial deviant eating behavior is becoming a severe psychosomatic disease, evolving for itself with denutrition and metabolic modifications promoting self-aggravation.

### Development of food preferences

Development of food preferences and eating behaviors is a complex and paradoxical process starting probably *in utero*, involving inherited taste characteristic and interactions with environmental factors such as familial eating behavior, peers influence, and socio-cultural context (Birch, 1999).

### Inherited characteristics

Study of gusto-facial expression in 2 years old infants defines ≪ hypoguesic ≫, ≪ normoguesic ≫, and ≪ hyperguesic≫ groups. Hyperguesic group develops more difficulties in food choices and in maternal feeding interaction (Chiva, 1982). Individual genetic sensitivity to the bitter taste of phenylthiocarbamide (PTC) or 6-n-propylthiouracil (PROP) determines food preferences and dietary habits. Thirty percent of occidental population is “non-tasters” vs. 50% “tasters” and 20% called “super-tasters.” Such a marker for individual differences in taste perception could influence food preferences (bitter and foods rich in antioxydants, sweetened and fat-containing foods, alcoholic beverages), and dietary behavior with subsequent links to body weight and chronic disease risk. There may be an inverse correlation between genetic sensitivity to PTC or PROP, BMI, and body fatness. The level of involvement of this phenotype mediated by the TAS2R38 gene in food regulation is still under debate. Other genetic bitter and non-bitter taste factors could play a role in food preferences including food aversion and deviant eating behaviors, such as the gustine gene which affects salivary ionic zinc concentrations—a trophic factor of taste buds (carbonic anhydrase VI). Otherwise, despite the fact that patients with anorexia nervosa often complain of disrupted smell and taste sense, there is no study concerning clinical population with eating disorders (Goldzak-Kunik et al., 2012).

### Parental feeding practices and sociocultural influences

Children eating behavior is precociously influenced by sociocultural environment, parents feeding practices and particularly maternal eating habits and food preferences. Negative affects at mealtime, eating conflicts, struggles with food, and unpleasant meals in early childhood are associated with higher risk for subsequent symptoms of anorexia nervosa. Maternal diet is one of the strongest predictor of childhood food consumption. Indeed, 15–20% of variation in children's difficulties could be associated with maternal feeding practices. A French study showed that extreme feeding practices as contingent, coercive or permissive style enhanced children's eating difficulties such as food neophobia, pickiness, low appetite, and low enjoyment in food. On the contrary, a flexible attitude, with repeated exposures to food, enhanced motivation to eat (Rigal et al., 2012). Parental excessive restriction and control of child feeding practices could limit the children eating autonomy and negatively influence their food choice, promoting in opposite reaction, consumption of “bad foods” including a diet rich in sugar, salt, and/or fat. Intrusiveness and other deleterious parental behavior, as not acknowledging the needs of the child, are also associated with higher risk of eating disorders (Gahagan, 2012).

### Parental eating disorders

Several studies have shown that offspring of mothers with eating disorders are at higher risk of developing psychopathology in childhood and adolescence. Maternal eating disorders influence very early the quality of infant feeding, starting *in utero* with more frequent prematurity, low birth weight (which are themselves risk factors for eating disorders), overweight, and hyperphagia (Micali et al., 2011). Women with high weight and shape concerns breastfeed less their newborns, and might influence future children food choice because of their own food restrictions during this breastfeeding period. Later on, conflicts and unpleasant remarks at mealtimes, intrusive and control feeding practices may promote child negative eating behaviors, opposition attitude with food refusal, or infantile anorexia (Micali et al., 2009). Longitudinal study comparing children of women with eating disorders with “non-exposed” children showed modified feeding behaviors with more adhesion to “health conscious/vegetarian” dietary pattern in the first group, less fat and more carbohydrates in children of women with bulimia nervosa, and more high energy–dense foods in children of women with a history of binge sub-type anorexia. These modifications of food choice may be associated with a further risk of developing weight concerns, overweight, and eating disorders in later childhood and adolescence (Easter et al., 2013).

### Premorbid overweight or obesity

There is no study concerning association between overweight or obesity in childhood and further episode of anorexia nervosa in adolescence. However, overweight or obesity in childhood are strongly associated with hyperphagia, even though incidence of other types of eating disorders is unknown. Even if anorexia nervosa and binge eating disorder differed on premorbid personality/behavioral problems, and family overeating, risk factors for bulimia nervosa are mainly shared with anorexia nervosa and binge eating. Premorbid BMI is positively correlated to binge eating disorders and bulimia nervosa during the course of anorexia nervosa episode (Nishumira et al., 2008). Overweight or obesity during pre-morbid period is more frequent in male adolescent with anorexia nervosa (30–40%). Otherwise, recent studies suggest that adolescents with a history of premorbid overweight or obesity may develop restrictive eating disorders, with severe weight loss, severe medical complications, and a poorer prognosis.

### Food neophobia/pickiness/ARFID

Even though studies do not provide firm conclusions concerning which problems are predictive, the presence of eating problems in early childhood or an eating disorder in adolescence confers a strong risk for an eating disorder in young adulthood (Kotler et al., 2001). Digestive troubles in neonatal period or in early infancy (severe gastro oesophageal reflux) or food aversions secondary to gastrointestinal disease with nausea and vomiting have been reported to have negative consequences on appetite and eating patterns. Food neophobia is a survival mechanism with rejection of unknown foods while picky/fussy eating, a frequent problem in childhood (8–50%). It is defined as a rejection of familiar food with consumption of an inadequate variety of food, and low intakes of vegetables, and fruits. Risk factors for a later development of eating disorders include picky eating and digestive problems with a significant stability over the 10-year span studied, beginning at ages 1–10 (Marchi and Cohen, 1990). Even if food neophobia and picky eating are two distinct entities, there is probably an overlap and both may be associated with a certain type of eating disorders. ARFID (Avoidant/Restrictive Food Intake Disorder in Children and Adolescents) is a new category of DSM-5 classification, including those with selective (picky) eating since early childhood. Common eating-specific behaviors and symptoms in the ARFID group contain food avoidance, loss of appetite, abdominal pain, and fear of vomiting. Patients with ARFID are younger than those with AN, more likely to present before age 12, and more likely to be male (Norris et al., 2014). In a recent study, patients with ARFID were significantly underweight with a longer duration of illness, more likely to have comorbid medical conditions, anxiety or mood disorder. The course of ARFID is unknown but it is possible that these children and adolescents would develop other types of eating disorder such as AN, BN, or EDNOS (Fisher et al., 2014).

### Conclusion

Anorexia nervosa takes root in vulnerability factors determining childhood feeding and eating behaviors bases. These susceptibility factors, including food preferences, inherited taste factors, environmental factors, and early inadequate food intakes may be impacted by modifications associated to the pubertal process, therefore untitled the “mismatching team.” Detection of particular traits of eating patterns or deviant ponderal history may indicate vulnerability and high risk for some toddlers or children to develop anorexia nervosa in adolescence.

## Model 7: anorexia nervosa (AN) is an attempt to preserve mental homeostasis

**Figure 7:**
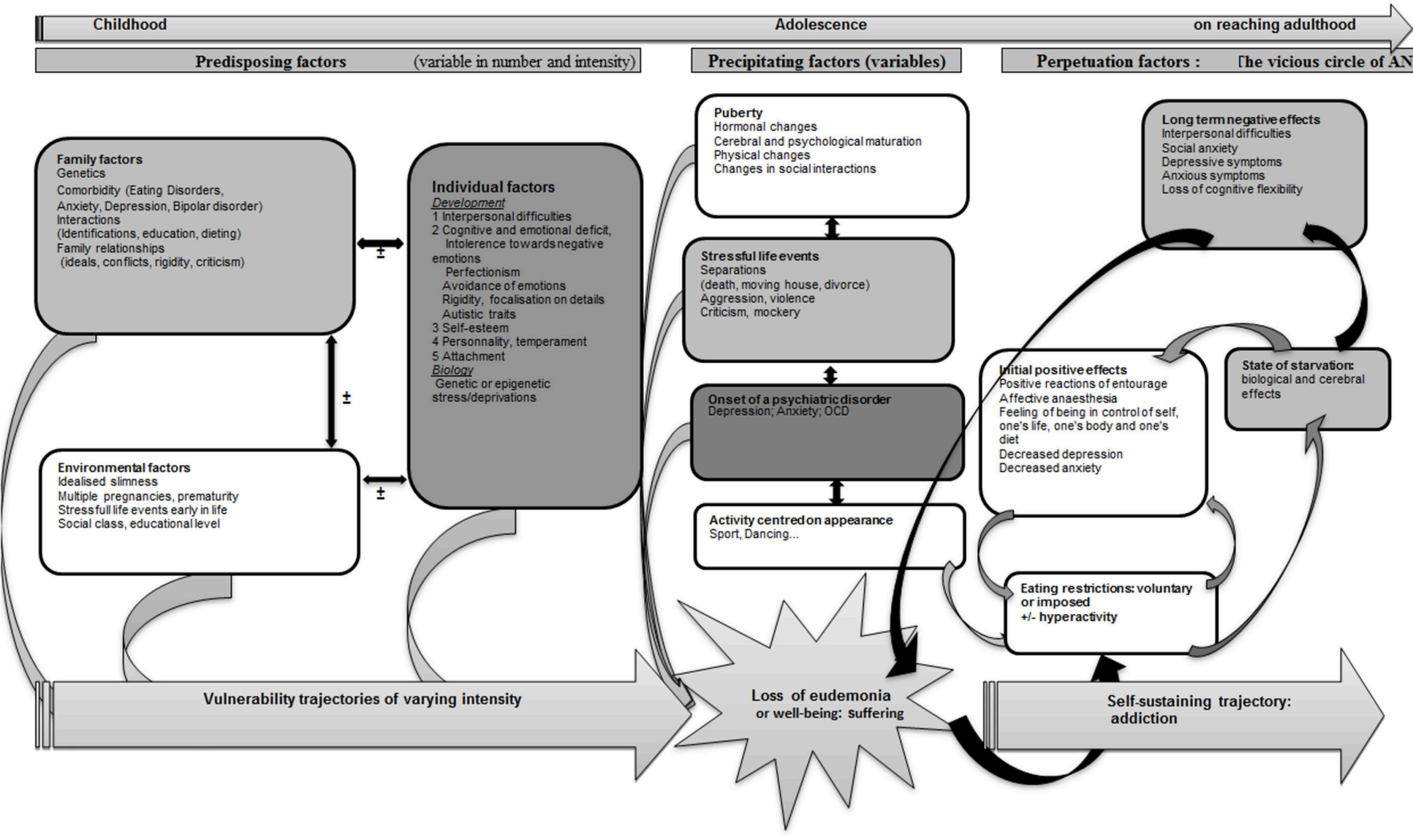
**A model of anorexia nervosa as an attempt to preserve mental homeostasis**.

AN can be considered as the result of an attempt to restore mental homeostasis that uses the bodily domain by way of restricted eating (Figure 7). Certain subjects, because of predisposing factors (Garner et al., 1993) could develop trajectories of vulnerability toward AN. This vulnerability could come to the surface on the occasion of precipitating elements leading to the loss of “well-being” or “eudemonia” as a result of individual factors (mental or somatic) or interpersonal factors (See diagram). The unsettled homeostasis, in a first stage, is restored by restricted eating, a process that could contribute to prolonging AN. Vulnerability trajectories and precipitating factors are liable to vary in nature and intensity from one subject to another, and would explain the variable clinical expressions in terms of age at onset, clinical symptoms, intensity of the disorder, duration of evolution, therapeutic response, or evolution to chronic illness, bulimia or other psychiatric disorders.

The predisposing elements contributing to an AN vulnerability trajectory are not all present at once in patients, as they assemble to form trajectories made up of numerous different vulnerability factors. These predisposing elements include (1) family factors, including genetic susceptibility to the disorder (Pinheiro et al., 2010; Trace et al., 2013), a psychiatric family history of eating disorders (Strober et al., 2000), anxious, depressive, or bipolar disorders (Bould et al., 2015), traits such as perfectionism or rigidity (Treasure et al., 2010), disturbed family interactions early in life (deprivation, violence—Jaite et al., 2012) or family interactions connected with eating habits (Hill and Franklin, 1998; Haycraft et al., 2014; Loth et al., 2014; Micali et al., 2014). Some (2) individual factors might also be involved, such as female gender (Connan et al., 2003), early exposure to stress, including intra-uterine stress (Connan et al., 2003; Treasure et al., 2010) via epigenetic regulation (Toyokawa et al., 2012) and disrupted personal development evidencing itself in affects, cognitions and interpersonal relationships (Connan et al., 2003). Depending on the concepts mobilized in different studies, these phenomena are evidenced in the form of personality disorders or dimensions (perfectionism, obsessive-compulsiveness, neuroticism, negative emotionality, harm avoidance, low self-directedness, low cooperativeness, low novelty seeking, and traits associated with avoidant personality disorders) (Cassin and Von Ranson, 2005), attachment disorders (Tasca and Balfour, 2014), emotional regulation disorders (Haynos and Fruzzetti, 2011), cognitive disorders (weak central control, rigidity) (Treasure and Schmidt, 2013), anxiety disorders (Raney et al., 2008; Touchette et al., 2011), and biological or genetic particularities (see for example Bailer and Kaye, 2011; Tremolizzo et al., 2014). (3) Environmental factors also play an important role, including stressful life events such as prematurity (Favaro et al., 2006), multiple birth (Raevuori et al., 2014), and social factors such as the ideal of slimness in society (Garner, 1993), social class (Godart et al., 2013), and educational level (Goodman et al., 2014).

The trajectories derived from these various elements remain to be characterized. They could roughly correspond to five types, dominated respectively by (1) obsessiveness and perfectionism, (2) major interpersonal difficulties verging on Asperger's syndrome, (3) anxiety, (4) mood disorders, recurrent unipolar depression, bipolar disorders, and (5) personality disorders interwoven with deprivation or violence in the early years.

The precipitating elements are mainly stressful life events (Connan et al., 2006; Schmidt and Treasure, 2006) or negative emotional states (Fox and Power, 2009), sometime in a setting of incipient psychiatric comorbidity (depression, anxiety, OCD, Godart et al., 2002, 2007), or most often associated with puberty and including hormonal factors (Klump, 2013) or psychological, physical, and relational factors.

The start of eating restrictions appears in various contexts: (1) the psychological dissatisfaction linked to a negative emotional state (poor self-esteem) is transferred to the body (Fox and Power, 2009) and triggers a will to restrict food intake linked to dissatisfaction with the body, (2) a slimming diet (as a result of weight gain, family pressures, peer group influence), (3) an intercurrent event—operation involving the digestive tract, (4) emotional state (via stress—Greeno and Wing, 1996—anxiety or depression) or a somatic pathology can trigger anorexia in its prime meaning (loss of appetite). However, whatever the previous vulnerability trajectory or the precipitating events, these subjects discover the positive effects of food intake restrictions (as has been described in the short term in depression, Fond et al., 2013), enabling a temporary return to eudemonia. This wellbeing is the result of biological modifications that may be caused by starvation, the decrease in anxious and depressive symptoms (Kaye et al., 2003), the feeling of having control over one's life, one's body, and one's diet, the sometimes positive feedback from the entourage, and in some cases affective anesthesia (Garner, 1993; Fox and Power, 2009).

In all cases, however, the balance obtained is fragile and short-lived, since the causes of the imbalance persist. Further to this, the lack of food (often compounded by hyperactivity), because it has biological and hormonal consequences, (leads to anxious and depressive symptoms Swenne and Rosling, 2010; Gauthier et al., 2014), interpersonal difficulties (Treasure and Schmidt, 2013), social anxiety (Coulon et al., 2009), and parental criticism linked to the patient's state (Treasure and Schmidt, 2013; Duclos et al., 2014) with which the subject tries to cope by the same means of food intake restriction, even intensifying food deprivation, in order to regain the same effect, which encloses the subject in the vicious circle of AN. In this model, AN can be considered as an addictive behavior in which the object of the addiction is the control exercised over one-self and one's hunger (Jeammet, 1991; Venisse, 1991; Goodman, 2008; Duclos et al., 2014).

The clinical profile observed by clinicians is composite, and depends on elements linked to the vulnerability trajectory (for example one of the five previously mentioned), to age at onset of the disorder (for instance pre-pubertal subjects exhibit growth and pubertal delay), adolescents are less often in denial of their thinness than adults (Couturier and Lock, 2006). It also depends on precipitating factors (for example if a depressive state precedes the disorder, suicidal ideas or psychomotor retardation can be present) and on the intensity and duration of food deprivation. We hypothesize that the severity of the AN evolution trajectory is also linked to the severity of vulnerability factors, to the intensity of the state of starvation and to AN prognosis: the more severe the difficulties experienced before AN onset, the more likely is the disorder to be severe and become chronic, and the greater are the mental disturbances compounding AN.

We propose a global, integrative clinical model of Anorexia Nervosa (AN), starting from models developed previously (Garner, 1993; Connan et al., 2003; Fairburn and Harrison, 2003; Schmidt and Treasure, 2006; Fox and Power, 2009; Herpertz-Dahlmann et al., 2011; Tasca et al., 2013; Treasure and Schmidt, 2013; Treasure et al., 2014) and from the concept of mental homeostasis, which postulates (Agnati et al., 2011) (1) that the concept of biological homeostasis can be transposed to the mental domain, (2) that any individual, from a psychological viewpoint, possesses a state of equilibrium called “the well-being of the human psyche” or “eudemonia,” and (3) that mental homeostasis sustains eudemonia by enabling a person to feel at ease with him/herself (emotionally and bodily) and with others (interpersonal relationships). Any event in one or other of these areas (emotional, bodily, interpersonal) that upsets this mental homeostasis will generate a state of “mental allostasis,” which is painful for the subject, who will struggle against it by way of adaptive processes to restore the earlier equilibrium. A person's mental homeostatic equilibrium is a dynamic balancing process that is variably vulnerable, depending on a developmental trajectory that is linked to family, individual and environmental factors.

## Conclusions

Just as for many of complex disorders, scientific evidences can lead to different heuristic models in AN. An efficient model should use validated risk factors to further define the potentially involved mechanisms of actions of anorexia nervosa. The predisposing elements contributing (see Model 7) to AN include (1) presence of a psychiatric family history of eating disorders, (2) presence of personal anxious, depressive or bipolar disorder, (3) different personality traits such as perfectionism or rigidity, (4) disturbed family interactions early in life (deprivation and violence), (5) family interactions connected with eating habits, but also (6) female gender, (7) early exposure to stress, including intra-uterine stress, multiple birth and prematurity, and (8) disrupted personal development. Social factors also have a role, such as (9) the ideal of slimness in society, (10) social class, and (11) educational level.

One of the specificity of anorexia is the presence of excessive physical exercises even though there is food deprivation or purging behaviors in underweight patients. An abnormality of the rewarding value in AN of food and exercise has thus been proposed as a core feature, which is supported by the role of endogenous opioids and dopamine pathways in the reward properties of food (see Model 1), and indirectly by an excess of specific polymorphisms of the genes coding for opioid and dopamine receptors or transporters in AN. An abnormal reward process could potentially involve more specifically a preference for delayed reward and/or reduced capacity to (as in controls) favor immediate reward. Such preference for delayed reward does give sense to avoid food (in order to lose weight) while starving, and not resting (while exhausted). Amphetamine-induced dopamine release indeed increased anxiety in individuals recovered from AN potentially explaining why food-related dopamine release might generate anxiety in AN patients vs. pleasure in healthy subjects (see Model 2). Recent evidence for a (marginal) role of a calcineurin gene derived from the largest GWAS performed up to now, support this view, as this gene independently explains part of human variations in endurance exercise capacity and tolerance (He et al., 2010a). Proposing that an abnormal reward process is central in AN is supported by the fact that plasma levels of ghrelin- an orexigenic hormone which usually induces food-motivated behavior- is increased in AN. As Ghrelin exerts pleiotropic effects, for example through heterodimers, including the modulation of the dopaminergic reward system, its role in AN is possible, and indeed supported by some genetic studies. But the neurons from the LHA send projections to the brain reward circuitry also contain other factors apart from Ghrelin such as the expressing orexins, MCH, and 26RFa (see Model 3). Orexin neurons in the LHA is for example strongly linked to preferences for cues associated with drug and food reward, and orexin injected into the VTA increases dopamine release in the accumbens nucleus (NAc). Even though the role of the MCH system has never been investigated in AN, it is interesting to note that the orexigenic effect of MCH is largely dependent upon its action in the NAc. 26RFa levels were also abnormal in patients with restrictive AN, as significantly increased all over the day compared to healthy subjects, and mapping of GPR103 mRNA (the receptor of 26RFa) in the brain revealed that the 26RFa receptor is also expressed in the VTA and the NAc. The orexigenic neuropeptides including ghrelin, orexins, and 26RFa are therefore clearly up-regulated in AN their orexigenic profile potentially reflecting an adaptive mechanism to promote food intake, trying –inefficiently- to counteract undernutrition, and potentially reinforcing dopamine-induced anxiety in the reward system of AN patients, and thus aversion to food.

The gut microbiome contributes to the risk and pathogenesis of malnutrition through nutrient metabolism and immune function, but has also shown specificities in patients with AN, such as a deviation of microbiota, and an increase of *M. smithii* (see Model 4) which are directly involved in energy efficiency. It is thus possible that some pre-existing changes in gut microbiota induce and/or participate to food restriction, for example through the frequently associated chronic constipation observed in AN. Even more precisely, some authors propose that food restriction, via increased intestinal permeability, may favor bacterial protein translocation from the gut, resulting in increased production of neuropeptide-reactive Igs, the bacterial heat-shock chaperone protein (ClpB) potentially acting as an anorexigenic α-MSH mimetic protein. As the levels and properties of Igs reactive with ghrelin were also altered in patients with AN (see Model 5), antigens could involve other neuropeptides.

Apart from the inheritance of genetic polymorphisms from parents to children, risk factors may also get from a generation to the other through parents/child interactions. Indeed, women with high weight, and shape concerns, breastfeed less their newborns (see Model 6). Later on, conflictive, intrusive and excessive controls of feeding practices promote child negative eating behaviors (references). Longitudinal study also showed that women with eating disorders are more frequently adopting “health conscious/vegetarian” dietary pattern. Furthermore, eating disorder in adolescence, such as food neophobia, pickiness, or ARFID (Avoidant/Restrictive Food Intake Disorder in Children and Adolescents) confers a strong risk for an eating disorder in young adulthood.

**Figure 8:**
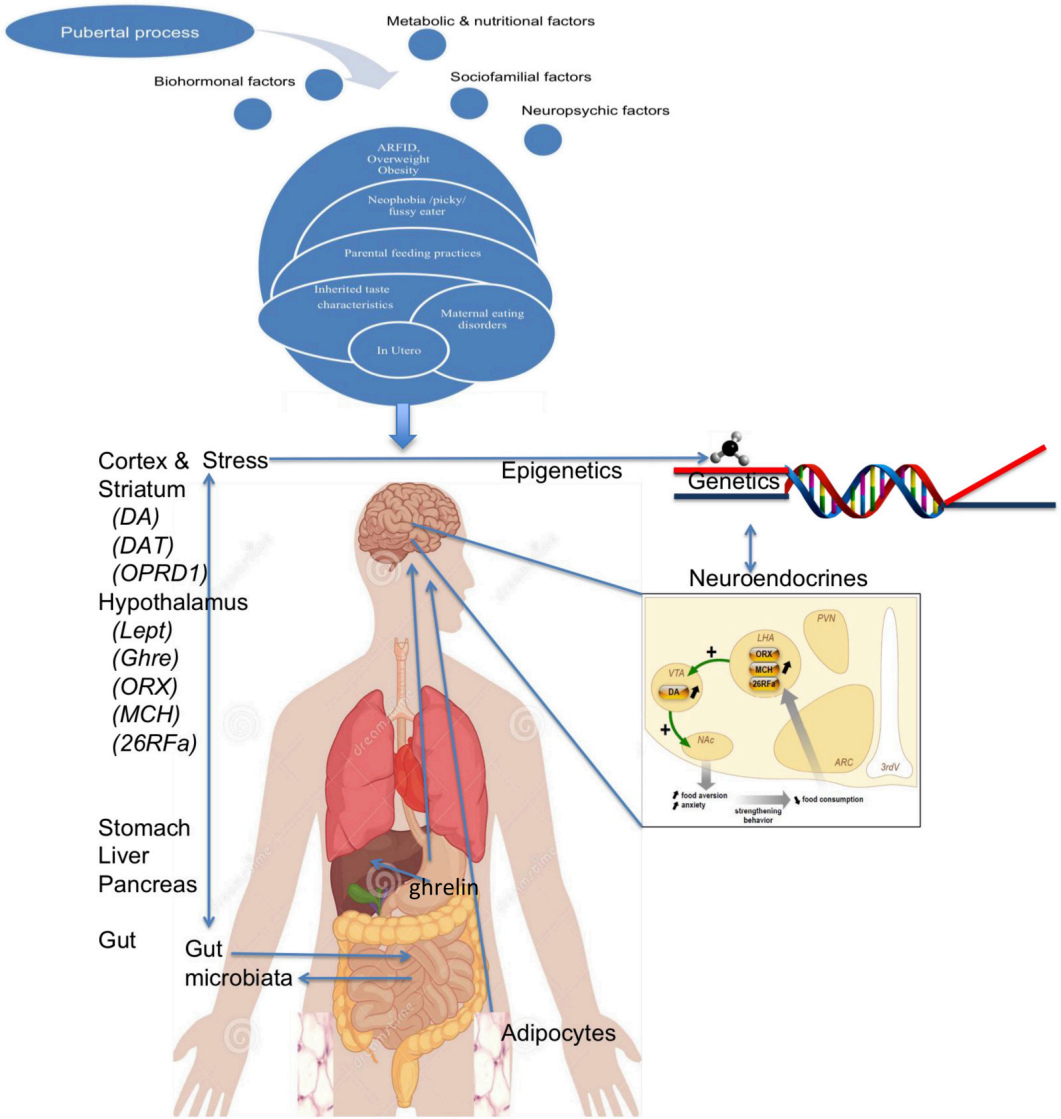
**A global model of anorexia nervosa**.

All together (Figure 8), we could propose that subjects genetically at risk and/or exposed to pathological feeding attitudes by their parents, may have early non-specific eating disorders that are usually unnoticed, such as traits related to food neophobia, pickiness or ARFID. In relation to other risk factors, such subthreshold difficulties could worsen in some patients (for example because of personal or familial psychiatric morbidity, perfectionism or rigidity traits or rigidity), and even develop anorexia nervosa when meeting triggering factors (such as stress around puberty), in a facilitating environment (wealth of food and high value of thinness). Throughout such pathway, food would progressively change its rewarding value, losing the positive value of satiety, or increased the positive feelings associated with weight loss or excessive exercise, potentially through an increased preference for delayed reward. Interestingly, such progression of the disease could be explained by the accumulation of risk factors (occurring at different times of life), but also by gene × gene interaction (two-hit hypothesis), epigenetic modifications (throughout a gene–environment interaction), and even via increased intestinal permeability which may lead to increased production of neuropeptide-reactive immunoglobulins. Even if some aspects are still speculative, they lead to two important conclusions. Firstly, many aspects developed herein are still very preliminary in anorexia nervosa, and could be much more important than initially thought (such as microbiota, epigenetic regulations, and immunity) when studied with more patients and more powerful technics. Secondly, proposing an integrative model of AN reinforces the idea that depicting the mechanisms of the disorder will need to study those different aspects together, all singular approaches would decrease the chances to cover all aspects of this complex disorder.

## Author contributions

JE wrote the introduction and conclusion with PG and reviewed the different parts of the article and figures. PG wrote the introduction and conclusion with JE and reviewed the different parts of the article and figures. CB, NC, JD, PD, MH, SF, NG, JM, NR, CR, VT, and OV participated in writing the article and designing of figures.

### Conflict of interest statement

The authors declare that the research was conducted in the absence of any commercial or financial relationships that could be construed as a potential conflict of interest. The reviewer VP declared a shared affiliation and previous co-authorship with author OV to the handling Editor, who ensured that the process nevertheless met the standards of a fair and objective review.
